# A novel scale to assess psychosis in patients with parkinson's disease

**DOI:** 10.1186/s40734-015-0024-5

**Published:** 2015-12-01

**Authors:** William G. Ondo, Sana Sarfaraz, MinJae Lee

**Affiliations:** Department of Neurology, Methodist Neurological Institute, 6560 Fannin, Ste 802, Houston, TX 77030 USA; Biostatistics/Epidemiology/Research Design (BERD) Core, Center for Clinical and Translational Sciences, The University of Texas Health Science Center at Houston and Division of Clinical and Translational Sciences, Houston, TX 77030 USA; Department of Internal Medicine, The University of Texas Medical School at Houston Center for Clinical and Translational Sciences, University of Texas Health Science Center at Houston, Houston, TX 77030 USA

**Keywords:** Parkinson’s disease, Psychosis, Hallucinations, Delusions, Scale, Metrics

## Abstract

**Background:**

Organic psychosis effects up to 70 % of patients with PD at some point yet no widely accepted scale for this entity exists.

**Methods:**

We developed a 10 question PD specific psychosis severity scale that we feel has good content validity. It asks about the presence, severity, frequency, and consequences of the hallucinations (visual, auditory, olfactory) and delusions.

**Results:**

Fifty different PD patients with psychosis and 25 PD patients without psychosis were included, and serial information was available in 21 of those encounters with psychosis. In psychosis subjects, results were normally distributed: mean 17.23 (SD = 6.30). In those without psychosis 14 % scored >0, mean 0.36 [range0-7]. The intra-rater, inter-class correlation coefficient was excellent (N = 21 pairs of observations seven days apart, ICC = 0.87). Inter-rater reliability (two different raters, N = 46 pairs) was outstanding for the entire group, ICC = 0.92). As expected visual hallucinations were most common (mean = 3.13). The presence of delusions was associated with greater total scores.

**Conclusions:**

This scale, specifically designed for PD psychosis is easy to administer and has impressive metrics.

**Electronic supplementary material:**

The online version of this article (doi:10.1186/s40734-015-0024-5) contains supplementary material, which is available to authorized users.

## Background

Psychosis in Parkinson’s disease (PD) affects up to 70 % of patients, and causes tremendous morbidity [1]. The phenotype is much different than psychosis associated with schizophrenia or delirium. Although many different hallucinations, illusions, and delusions are reported in PD, the majority of episodes are visual hallucinations (usually “benign” content of silent people or animals), and persecutory/infidelity delusions, which are usually more problematic [1].

Despite the prevalence and importance of PD psychosis, no widely accepted scale exists. A Movement Disorder Task Force evaluated scales used to assess psychosis in PD, and concluded that a novel PD specific scale is needed [2]. Several small scales designed specifically for PD were felt to be insufficiently inclusive or poorly validated [3, 4], whereas better known and validated psychosis scales were not designed for PD psychosis and were felt to have poor content validity [5–7].

The lack of a disease specific severity scale has probably hampered efforts to test treatments for PD psychosis. To this day only clozapine and pimavanserin have compelling published efficacy data [8, 9]. To better quantify PD psychosis and aid in future therapeutic trials, we designed a PD specific psychosis scale and undertook psychometric evaluations.

## Methods

The main goals for this scale development were content validity (based on the most common psychosis symptoms in the literature and experience, as there is no gold standard scale to compare), ease of use (10–15 minutes), inter-rater validation to include both physicians and non-physicians, and intra-rater validity measured over time. It is not a specifically designed quality of life scale, although questions 6–10 investigate how the psychosis would likely effect quality of life (insight, affective consequences and actions, and impact on family), nor is it designed as a screening tool. The questions evolved over 10 years and included patient and family input. Earlier non-validated versions of the scale have been used in previous clinical trials [10, 11].

The study received a waiver from full consent from the University of Texas Health Science Center Institutional Review Board. All PD patients were recruited from a movement disorder center. We included PD patients with psychosis, based on a rating of ≥1 on UPDRS question #2, as determined by interview with the investigator, and a comparator group of PD patients without psychosis, score = 0. There were no formal exclusion criteria other than complete inability to participate. We intentionally allowed subjects with clinical dementia but in all cases motor symptoms preceded cognitive symptoms by more than a year [12]. Dementia was diagnosed if patients had a chart documented diagnosis of dementia and/or were taking acetylcholinesterase inhibitors for dementia. All consecutively seen psychosis patients seen in our tertiary referral center were included, but the control group was a convenience sample from the same clinic seen over the same period.

**Fig. 1:**
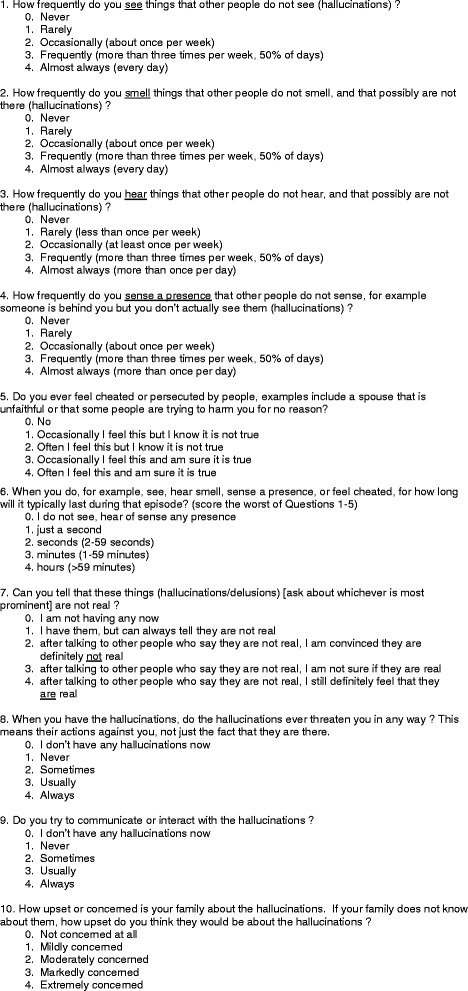
Actual Scale

The first five questions identify the type of hallucination (visual, auditory, olfactory, sense of presence) or delusion [Fig. 1: Scale]. The second five questions further quantify the intensity, frequency, insight and impact of the worst psychotic feature on the life of the patient and family. The source of the information (patient/family/both) is documented. Since insight varies among patients, some latitude in question semantics is allowed and we did not include a specific question on how the psychosis affects only the patient’s quality of life. [Additional file 1: Instructions] The final answer (0–4 for each question) is the opinion of the interviewer. It is not self-administered.

The patients were interviewed by an experienced physician and an inexperienced coordinator who had just started working with PD patient, at least 15 minutes apart (inter-rater assessments). Physician interview preceded the “inexperienced” interview in all cases. Intra-rater reliability (test-retest) was also tested at a second point in time 7+/−3 days after the first administration, in patients who did not require therapeutic intervention (change in medications) prior to then. This was done by a single interviewer (WO).

**Fig. 2:**
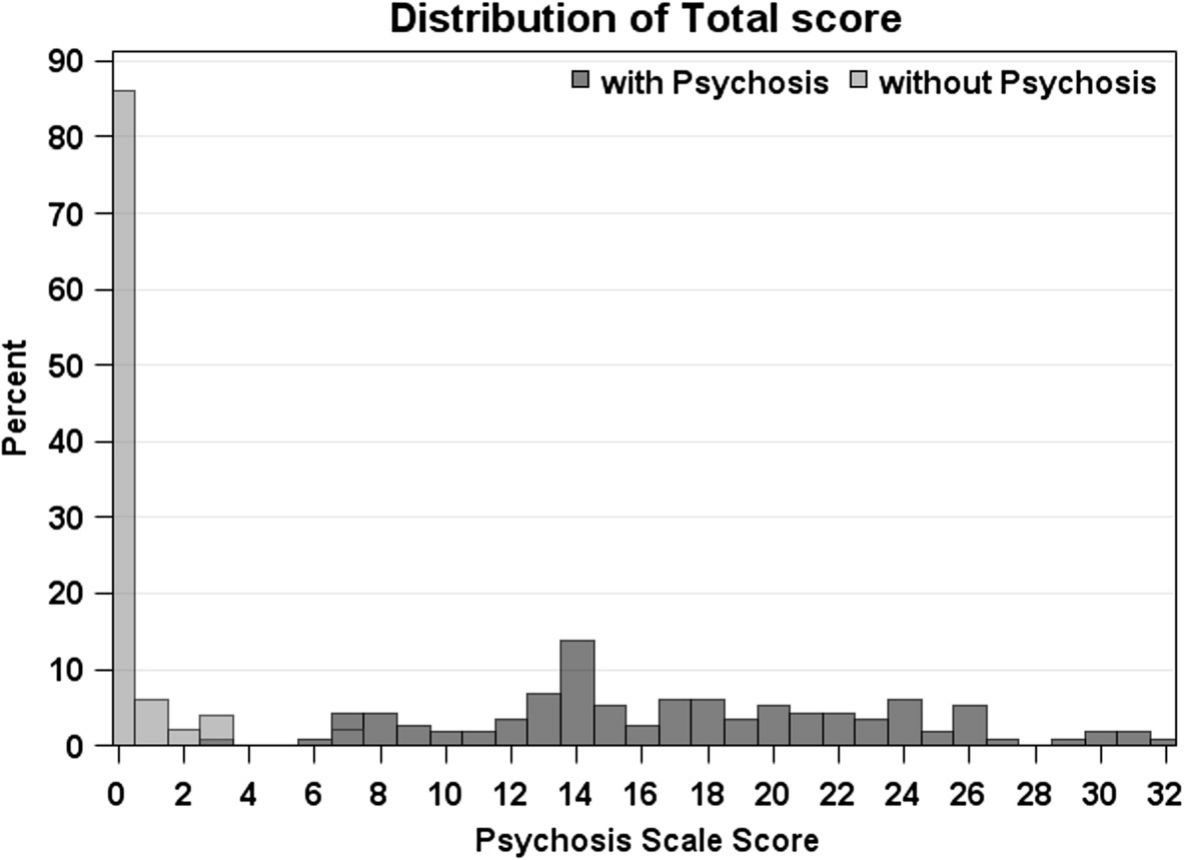
Histogram of Total Scores *N* = 100 scales with psychosis and 50 scales without psychosis

The range of score for each specific question is 0–4 and the total score simply adds all 10 questions. Weighted kappa statistics were calculated for inter and intra-rater reliability on the scale ranged 0–4 for each question, and intra-class correlation coefficients were calculated for inter- and intra-rater reliability on total score and individual questions. We used the mixed effect model to account for correlation within subjects. Poisson and linear model were conducted for patients with/without psychosis and with psychosis respectively because the total score for those with psychosis is normally distributed but not for those with non-psychosis [Fig. 2]. Descriptive statistics are also presented. Analyses were performed using SAS 9.3 (SAS Institute Inc, Cary, NC) at a statistical significance level of 0.05.

## Results

On average the questionnaire required a 10 min interview and family input was obtained in 84.8 % of all interviews. Inter-rater testing was completed on 46 different subjects with psychosis and 25 PD patients without psychosis. One subject was excluded because psychosis status was not definitively marked, leaving 49, and three psychosis subjects had intra-rater serial data, but not inter-rater data. The mean age for all 75 subjects (51 male) was 70.0 ± 10.8 years and duration of PD motor features was 9.4 ± 5.1 years. Dementia was diagnosed in 26 of the 75 total subjects (34.7 %). The PD without psychosis and PD with psychosis groups had similar age (68.8 ± 9.1 vs 70.6 ± 11.6 years), duration of PD 8.5 ± 5.0 vs 9.9 ± 5.1 years), percent that were male (68 % vs. 70 %), and percent that were demented (36.0 % vs. 34.0).

For those with psychosis, the results were normally distributed with a mean score of 17.23 ± 6.30, [range: 3–32]. Only 7 of 50 questionnaires from subjects without psychosis scored greater than 0, and only 1 scored >3 [Fig. 2]. The inter-rater reliability was excellent for the entire group (71 pair including psychotic and non-psychotic subjects, ICC = 0.92). For just those with psychosis (46 pairs) the ICC was 0.87. The intra-rater, inter-class correlation coefficient (time 1 vs. time 2 with same administrator) was excellent, N = 21 pairs of observations, ICC = 0.88. As expected, visual hallucinations were most common (mean = 3.13), followed by sensing a presence (mean = 2.06), auditory (mean = 1.16), and olfactory (mean = 0.29) [Table 1]. A delusion (question #5) was scored as >0 in 36/100 PD psychosis questionnaires. The total score in these cases with any delusion was much higher than those with pure hallucinations without delusions (mean = 22.51 vs. 14.49, *p* < 0.001, powered by higher responses in questions #6–10 (mean of 12.53 vs 7.73, *p* < 0.001).

**Table 1:** Summary of Data

	Mean Score in Psychosis Subjects, mean (SD)	Number of questionnaires in Control Subjects with a score of >0 (out of 50 assessments)	Inter-Rater Reliability (95 % CI)^a^		Intra-Rater Reliability (95 % CI)^b^
			All (*N* = 71)	Psychosis patients (*N* = 46)	Psychosis (*N* = 17)
1. Frequency Visual	3.13 (0.98)	1	0.85 (0.79, 0.91)	0.62 (0.47, 0.77)	0.51 (0.23, 0.80)
2. Frequency Olfactory	0.29 (0.65)	2	0.85 (0.70, 1.00)	0.84 (0.66,1.00)	0.66 (0.47, 0.86)
3. Frequency Auditory	1.16 (1.50)	0	0.76 (0.66, 0.85)	0.72 (0.60, 0.83)	0.68 (0.51, 0.85)
4. Frequency Presence	2.06 (1.41)	4	0.79 (0.71, 0.87)	0.70 (0.58, 0.82)	0.72 (0.54, 0.90)
5. Delusion Assessment	0.97 (1.41)	2	0.64 (0.47, 0.81)	0.59 (0.40, 0.45)	0.65 (0.33, 0.97)
6. Duration of Psychosis	2.66 (0.90)	4	0.82 (0.73, 0.90)	0.59 (0.42, 0.77)	0.46 (0.15, 0.77)
7. Insight	2.03 (0.97)	2	0.75 (0.65, 0.86)	0.58 (0.41, 0.76)	0.65 (0.48, 0.82)
8. Threatening	1.34 (0.60)	0	0.82 (0.73, 0.91)	0.62 (0.43, 0.80)	0.77 (0.47, 1.00)
9. Interaction	1.71 (0.82)	0	0.81 (0.73, 0.90)	0.62 (0.45, 0.80)	0.37 (0.07, 0.66)
10. Family Concern	1.90 (1.42)	0	0.86 (0.78, 0.94)	0.80 (0.68, 0.92)	0.86 (0.73, 0.99)
TOTAL	17.23 (6.30)	7	0.92 (0.89, 0.94)	0.87 (0.80, 0.92)	0.86 (0.80, 0.91)

Weighted kappa statistics for each of 10 specific questions and intra-class correlation coefficients (ICC) for total score were shown as Inter- ^a^and Intra-rater ^b^reliability

## Discussion

We report very good intra-rater reliability and excellent inter-rater reliability on a 10 question scale designed specifically for PD associated psychosis. Several content features warrant comment. We did not include a separate question on illusions because subjects often have difficulty differentiating these from true hallucinations so it could be “double scored”. We instruct that illusions be included with hallucinations (almost always visual). We did not include tactile hallucinations. These have been reported [13, 14], but in our experience are essentially impossible to differentiate from “actual” sensations, which are very common in parkinsonism, and do not respond similarly to interventions to reduce psychosis (dopamine dose reduction, addition of anti-psychotic medications), suggesting they are intrinsically different and perhaps not best thought of as “hallucinations”. It is also our opinion that they do not contribute much to morbidity. “Passage” (very brief) hallucinations/illusions are differentiated from more prolonged visual hallucinations in the duration score. Delusions are usually the most problematic psychosis. We have only a single question on amount of delusions, compared to four regarding different hallucinations, but hypothesized that the final five questions regarding how the psychosis affects the patient would score higher on subjects with delusions, and compensate for the single delusion question. Even if question #5 (delusions) is excluded from the total score summation, patients who scored >0 on #5 had significantly higher scores (*p* < 0.001). The last question regarding impact on family was included to account for the poor insight many patients possess, the fact that families are often more disturbed by psychosis than the patient, and because some of the major consequences of psychosis, such as nursing home placement, are often determined by the caregivers. Since patients themselves have markedly variable insight into their own hallucinations, asking impact of their own psychosis would be difficult to quantify. We also did not exclude patients with varying degrees of cognitive impairment, who may further require family input. We did not formally assess cognition at time of assessment so can’t statistically compare “demented” vs “not-demented” subject results.

Our scale has several potential weaknesses. Data on test-retest reliability was skewed towards subjects with less severe psychosis, as more severe subjects required immediate interventions, and therefore could not be reassessed for this purpose. We did not compare our results to any validated general psychosis study (content validity) because their content was not designed for the PD psychosis phenotype so any subsequent interpretation of “content” validity would have limited utility. Content validity was excellent based on UPDRS psychosis question (mean 17.23 for score >0 vs. 0.36 for 0) We did not formally assess sensitivity to change with intervention, although several subjects started on clozapine showed marked reduction in scores (data not shown).

The final 10 question set was created over a decade and included patient and family input, however they were not formally culled from a larger set and did not undergo cognitive pre-testing. We included subjects with dementia, as this is common in hallucinating patients, so family input was absolutely necessary in this group, as demented subjects could not understand or respond to some of the questions by themselves. No subject had psychosis in the absence of dopaminergic medications, but we did not attempt to exclude the clinical diagnosis of dementia with Lewy bodies, except by onset of motor vs. cognitive symptoms. Importantly, the questionnaire is not meant to be self-administered and some interpretation of response is needed by the interviewer (discussed more fully in the Additional file 1: Instructions), nor is it meant to be a screening tool to diagnose psychosis. Future research could formally assess sensitivity to treatment response, correlations with other scales assessing quality of life, other scales for psychosis, formal comparison of demented vs. non-demented patients, comparison of patient vs family scores, and neurophysiology correlates.

## Conclusion

We feel this scale offeres very good content valisity, inter- and intra-rater reliability and ease of use.

## Additional file

Additional file 1:
**Instructions for completing the psychosis scale.** (DOCX 17 kb)
