# Supplementary material for: A novel scale to assess psychosis in patients with parkinson's disease
Source: J Clin Mov Disord. 2015 Dec 1;2:17. doi: 10.1186/s40734-015-0024-5 (PMC4711049; doi:10.1186/s40734-015-0024-5)
Supplement: Additional file 1: — Instructions for completing the psychosis scale. (DOCX 17 kb) [file 40734_2015_24_MOESM1_ESM.docx]

**Appendix:** Instructions for Hallucination Questionnaire

General Consideration:

Questions about psychosis have intrinsic difficulties. Foremost is the fact that the subject may have no insight regarding the hallucination/delusion and insist they are real. Therefore, “hallucinations” and “delusions” are determined by the opinion of the examiner, and the examiner may substitute the actual hallucination/delusion (i.e. the people sitting on the couch) instead of using the word “hallucination/delusion” when administering the questionnaire. Often, this will involve input from the patient and family members. The score should be that which the interviewer feels is most credible. Often subjects will minimize the problem.

Specific questions:

Questions 1-4 ask about specific sensory modalities. These questions refer to the past week.

Touch is not included because it is felt this is a very rare hallucination, whereas abnormal sensations (pain, numbness etc.) are very common in PD, and there is no way to truly differentiate these.

Regarding visual hallucinations: illusions (misinterpretation of objects) is included and scored similarly to if it were a true hallucination. “Passage hallucinations” are simply captured in the duration question #6.

Regarding audio hallucinations: We also caution that audio hallucinations are less common. Subjects often report that their visual hallucinations are talking, but with further questioning, it is rare that they are actually heard, but are rather silent, therefore this would not count as an audio hallucination. If the subject does clearly hear voices of the visual hallucinations, or any other sound thought to be a hallucination, it would count as an audio hallucination.

Regarding sense of presence hallucination: This must be different from their visual or other hallucinations. If they sense a presence of someone they are also seeing (visual hallucination), this does not count as a separate sense of presence.

Question 5 addresses delusion. It cites the two most common delusions (persecution and spousal infidelity) but could refer to others i.e. somatic, religiosity, etc. Again when discussing this, the actual delusion i.e. “wife is cheating”, can replace the word “delusion”.

Questions 6-9 determine the intensity, clinical significance/consequences, and insight of the hallucinations or delusions. They are scored for whichever hallucination/delusion (question 1-5) is most severe. If the actual score between the first five questions is a tie, it applies to whichever the interviewer determines is the most severe, and would result in the greatest score for questions #6-9.

Question 6 is the duration of the typical individual episodes of hallucinations. They are not additive throughout the day.

Question 7 queries how strongly their beliefs are that the hallucinations/delusion are real and how readily they can be allayed or dissuaded by others.

Question 8 queries if the hallucinations are threatening. It is specifically asking about the actions of the hallucinations (weapons, menacing faces etc.), not just the fact that they are in the subject’s house. A visual hallucination that is benignly sitting on the couch not interacting with the subject should not be scored as threatening even if the subject is very concerned about this.

Question 9 queries the consequences of the hallucinations/delusions.

Question 10 is largely a gestalt of subjective concern regarding the problem. If there is no family involvement, the subject is asked to speculate how concerned he thinks their family would be.
